# Sequential Quadriplex Real-Time PCR for Identifying 20 Common *emm* Types of Group A *Streptococcus*

**DOI:** 10.1128/JCM.01764-20

**Published:** 2020-12-17

**Authors:** Srinivasan Velusamy, Katherine Jordak, Madeline Kupor, Sopio Chochua, Lesley McGee, Bernard Beall

**Affiliations:** a Respiratory Diseases Branch, Centers for Disease Control and Prevention, Atlanta, Georgia, USA; b Oak Ridge Institute for Science and Education, Oak Ridge, Tennessee, USA; c IHRC Incorporated, Atlanta, Georgia, USA; Washington University School of Medicine

**Keywords:** group A *Streptococcus*, M protein, vaccine, *emm* typing, multiplex real-time PCR

## Abstract

We developed a sequential quadriplex real-time PCR-based method for rapid identification of 20 *emm* types commonly found in invasive group A *Streptococcus* (iGAS) strains recovered through the Centers for Disease Control and Prevention’s Active Bacterial Core surveillance. Each *emm* real-time PCR assay showed high specificity and accurately identified the respective target *emm* type, including *emm* subtypes in the United States. Furthermore, this method is useful for rapid typing of GAS isolates and culture-negative specimens during outbreak investigations.

## Abstract (precis)

We developed a sequential quadriplex real-time PCR-based method for rapid identification of 20 *emm* types commonly found in invasive group A *Streptococcus* (iGAS) strains recovered through the Centers for Disease Control and Prevention’s Active Bacterial Core surveillance. Each *emm* real-time PCR assay showed high specificity and accurately identified the respective target *emm* type, including *emm* subtypes in the United States.

## INTRODUCTION

Group A *Streptococcus* (GAS) causes both mild infections (pharyngitis, anitis, and impetigo) and invasive diseases (acute rheumatic fever, rheumatic heart disease, and necrotizing fasciitis) worldwide (1). In the United States, nearly 24,000 invasive GAS infections were estimated in 2018 (https://www.cdc.gov/abcs/reports-findings/survreports/gas18.html). GAS is an important cause of severe, life-threatening illness among the elderly population, particularly those individuals residing in long-term-care facilities (LTCFs) (2) and skilled nursing facilities (SNFs) (3). Currently, multiple vaccines are in early stages of development to protect against this medically important pathogen (4).

The M protein, encoded by the *emm* gene, is a major GAS virulence factor traditionally targeted in serotyping GAS isolates (5). In-house antisera and agglutination methods were used to identify up to 80 classical *emm* serotypes (6). More than 25 years ago, a method for deducing M serotypes based on the 5′ variable region sequences of *emm* genes was described (7). This method was subsequently standardized at the CDC and became widely used worldwide (5). To avoid confounding with *emm*-like genes, the CDC’s current whole-genome-sequence-based approach relies upon identification of gene sequence that is linked to the 5′-situated primer 1 (7). This reference method, which relies upon a 180-bp *emm* sequence encoding the surface-exposed M protein N-terminal 50 residues and 10 residues of conserved signal peptide (8), has identified over 250 *emm* types and 2,200 subtypes (https://www2.cdc.gov/vaccines/biotech/strepblast.asp). Recently, a new reverse primer (CDC3) was designed and validated to replace the less specific primer 2 of the first *emm* typing scheme (7), with a resultant improvement of amplification specificity (9). Sequencing and whole-genome sequencing (WGS)-based methods (2, 3, 5, 10) are relatively costly compared to PCR approaches, especially for resource-limited settings, and are not always suitable for outbreak situations, in which fast results are important.

Here, we describe a TaqMan-based quadriplex real-time PCR strategy to rapidly identify 20 *emm* types (*emm1*, -*2*, -*3*, -*4*, -*6*, -*11*, -*12*, -*28*, -*49*, -*59*, -*75*, -*76*, -*77*, -*81*, -*82*, -*83*, -*87*, -*89*, -*92*, and -*118*) that were the most common among invasive GAS (iGAS) recovered through CDC’s year 2015 invasive GAS surveillance (11).

## MATERIALS AND METHODS

### Bacterial isolates.

All bacterial isolates were cultured on Trypticase soy agar (TSA) with 5% sheep blood plates at 37°C with 5% CO_2_. Genomic DNA was extracted from isolates using the Qiagen DNA minikit (Qiagen, Valencia, CA) as previously described (12), with a prelysis step. Briefly, a loopful of bacteria grown overnight from a blood agar plate was suspended in 50 μl of lysis solution (lysozyme, 0.08 mg/ml, and mutanolysin, 150 U/ml) and incubated at 37°C for 1 h, followed by steps described in the kit manufacturer’s user manual.

A total of 253 bacterial isolates were used in this study to validate the typing scheme. The specificity and sensitivity of each of the five quadriplex assays were validated with respective *emm* type GAS isolates collected from Active Bacterial Core surveillance (ABCs). Twenty isolates of known S. pyogenes *emm* types, including *emm1*, -*2*, -*3*, -*4*, -*6*, -*11*, -*12*, -*28*, -*49*, -*59*, -*75*, -*76*, -*77*, -*81*, -*82*, -*83*, -*87*, -*89*, -*92*, and -*118*, were used in assay optimization. Additionally, 189 isolates representing 179 subtypes of 80 *emm* types, 4 nontypeable GAS, and 6 group G streptococci (GGS) (Streptococcus dysgalactiae subsp. *equisimilis*) (6 subtypes of 5 *emm* types) were tested (see Table S1 in the supplemental material). The assays were further challenged for specificity with 44 non-S. pyogenes streptococcal and nonstreptococcal isolates representing 41 species, including the streptococci Streptococcus acidominimus, S. agalactiae, S. bovis, S. canis, S. didelphis, Streptococcus dysgalactiae subsp. *dysgalactiae*, S. dysgalactiae subsp. *equisimilis*, S. entericus, Streptococcus equi subsp. *equi*, Streptococcus equi subsp. *zooepidemicus*, S. gordinii, S. hyointestinalis, S. hyovaginalis, S. iniae, S. intermedius, S. milleri, S. mitis, S. mutans, S. oralis, S. ovis, S. parauberis, S. phocae, S. pluranimalium, S. pneumoniae, S. porcinus, S. salivarius, S. sanguinis, S. sinensis, S. thoraltensis, S. uberis, S. urinalis, and S. pseudopneumoniae (*n* = 4), as well as Enterococcus faecalis, Enterococcus faecium, Haemophilus influenzae type b, Neisseria meningitidis, Staphylococcus aureus, Staphylococcus epidermidis, Escherichia coli, and Pseudomonas aeruginosa. Streptococcal species were from the CDC reference collection and were identified using standard approaches (13).

### Real-time PCR.

The *emm* type-specific 5′ region for each *emm* type was selected by aligning all the available subtypes of each *emm* type from the CDC’s *emm* database (ftp://ftp.cdc.gov/pub/infectious_diseases/biotech/tsemm) using CLC Genomics Workbench 10.0. The oligonucleotides for individual *emm* types were designed by using PrimerQuest software (https://www.idtdna.com/pages/tools/primerquest) to capture all the subtypes in each *emm* type. The primers and probes with different reporting dyes (6-carboxyfluorescein [FAM], 6-carboxy-2,4,4,5,7,7-hexachlorofluorescein [HEX], 6-carboxy-X-rhodamine [ROX], and 1,1'-bis(3-hydroxypropyl)-3,3,3',3'-tetramethylindodicarbocyanine [Cy5]) and appropriate quenchers were synthesized at the CDC Biotechnology Core Facility. The oligonucleotide sequences with their chemistry and optimal concentrations are provided in Table 1.

**TABLE 1:** Oligonucleotides used in the multiplex real-time PCR *emm* typing assays

Name	Oligonucleotide sequence (5′–3′)	Optimal concn (nM)
Emm1-F	GTGATGGTARTCCTAGGRAAGTT	400
Emm1-P	FAM/TRCGGGA-BHQ1dT-TGTTTGCTGCAAGADR/Spc6	100
Emm1-R	CATTGCATTCTCTAATCTCGCTTT	400
Emm2-F	CAGTTAAGGCGAACAGTAAGAACCC	200
Emm2-P	Cy5/AAATTAAG-BHQ2dT-GAAGCAGAATTACATGACAAA/Spc6	200
Emm2-R	GCTCTTCTTCAACTTTATCTAATTTCTCGAATA	200
Emm3-F	GCAGACAGTAAAGGCAGATG	300
Emm3-P	ROX/TRGGAGTGT-BHQ2dT-AATGVAGAGTTTYCTAGRCA/Spc6	300
Emm3-R	CCTGATHTAACAAGTTCTCAGTTTC	300
Emm4-F	CTGRTTCAGCGYGGAACT	300
Emm4-P	Cy5/CTRAARAA-BHQ2dT-ATAACGYGTTASTTVASGAAAATGAGG/Spc6	200
Emm4-R	TYCACGTTCTACCTTGARC	300
Emm6-F	TATTCGCTTAGAAAATTAAAAACAGG	300
Emm6-P	Cy5/TGAAGTTAG-BHQ2dT-GCAAGAGYGTTTYCTRGGG/Spc6	100
Emm6-R	TGTTAASYTGTCATTATTAGCTT	300
Emm11-F	GGCTTTGCAAAYCAAACAGAAG	300
Emm11-P	HEX/TYCTAAAGS-BHQ1dT-AMAAACGTGAKCGCA/Spc6	300
Emm11-R	TTCATCCCATAGCGAATTATATAGG	300
Emm12-F	AGATCATAGTGATTTAGTCGCAGAA	300
Emm12-P	ROX/TTTGVAAGAC-BHQ2dT-GAAACRGCDTTCAG/Spc6	300
Emm12-R	ATAGTATTGCTGAAGGTAGDDTT	300
Emm28-F	GRCTTYTGCTAATGGARCTGRT	400
Emm28-P	FAM/AGYTGM-BHQ1dT-GYATACAACACATTGCTTACTG/Spc6	100
Emm28-R	ACTCATCWCYGAGTTTCTCATGTT	400
Emm49/151-F^*a*^	TGCGGAGAATAACGTGTMTA	400
Emm49/151-P	HEX/CGTATAGY-BHQ1dT-CTTTTTCTCTTCTTGCAACGC/Spc6	300
Emm49/151-R	CCTATTCTTTCTAGATATTCTCCGT	400
Emm59-F	CTTTGCAAACCAAACAGAAGTTAAG	200
Emm59-P	FAM/AAATACGA-BHQ1dT-GYATTGACTAATGAGAATAAGTCT/Spc6	100
Emm59-R	TAATTTAAATAGTTATCTCTCTCTCTTCTAA	200
Emm75-F	TATGAAGCAYRATACAAAGCATGG	300
Emm75-P	FAM/AATTA-BHQ1dT-AGAARGACCTTAGATAAGTTTAATAC/Spc6	100
Emm75-R	CTAATCTCGTAGTCTTACCTTGCTCA	300
Emm76-F	TGCAAACCAAACAGAAGTTAAGG	300
Emm76-P	ROX/TGASCTACAGGC-BHQ2dT-GAACATGATAAGYT/Spc6	300
Emm76-R	GTTCAGYCAATAGCTCMTYAT	300
Emm77-F	TGCAAACCAAACAGAAGTTARGG	300
Emm77-P	ROX/AGTGATGCA-BHQ2dT-CTGAACCTACAGAAACCC/Spc6	200
Emm77-R	AAGGTCTGTAATGCGGTTATGT	300
Emm81-F	TGCAAACCAAACAGAAGTTAAGG	300
Emm81-P	HEX/TGCGGG-BHQ1dT-YCAGAAGARAATGTACYG/Spc6	300
Emm81-R	TTSAAGTTTGGCTATGTATKTCCG	300
Emm82-F	ATCACTGAGGCAGGTGTATCTA	300
Emm82-P	Cy5/TGGAAGAG-BHQ2dT-ARGTTTGATGCAGAGCA/Spc6	300
Emm82-R	TTTCAAGCTCGTTTGCTCTATTC	300
Emm83-F	CCAGACAGAAGTTAAGGCTGATAAC	400
Emm83-P	Cy5/CAATGCAG-BHQ2dT-AACACAAGRGSRCRC/Spc6	200
Emm83-R	TCCATTTCATGTAACAAATTCTGAAGC	400
Emm87-F	CYAGAGAAGTARCCARCGAA	200
Emm87-P	FAM/TTGGYTGC-BHQ1dT-TCARTGYGGAAGAAA/Spc6	200
Emm87-R	CTTTCCTTCTATTTCAGAGTAATCC	200
Emm89-F	TAAGGCGGACAGTGACAAT	300
Emm89-P	HEX/TGTCTCTG-BHQ1dT-CRAAGATAATGAAARAGAATTACATAACAVA/Spc6	300
Emm89-R	CATCTATTTTGTCTAGATGTTCTCCT	300
Emm92-F	GATGACCGGAGCGTTTCTAC	200
Emm92-P	HEX/AATAGTGG-BHQ1dT-AGCGTGAGCACAYCA/Spc6	300
Emm92-R	AGCTCACCATGTTTAGCCAATA	200
Emm118-F	GCGGACAGTAACGCG	300
Emm118-P	ROX/TCTAGCGT-BHQ2dT-GCAAAGCTATACAACCAAAT/Spc6	200
Emm118-R	CTCCGTTTTTATCTGTAAGATCG	300

a Assay targets both types *emm49* and *emm151*.

Optimal concentrations for primers and probes were determined by using SYBR green and TaqMan real-time PCR methods, respectively. Optimal concentrations of each oligonucleotide set were optimized with various concentrations (100, 200, 300, 400, and 500 nmol/liter) of each oligonucleotide against 10-fold dilutions of DNA from targeted *emm* type isolates in a Stratagene Mx3005P real-time PCR instrument (Agilent, Santa Clara, CA). The concentrations were optimized to obtain the highest DNA dilution yielding a cycle threshold (*C_T_*) value of ≤35. Lower limits of detection for each oligonucleotide set were determined in both singleplex and multiplex formats using 10-fold serial dilutions of the targeted *emm* type control DNA. In total, 20 individual assays targeting 20 *emm* types were grouped into five quadriplex reactions (Table 2) based on their distribution in the United States (10). The reaction mixture (25 μl) contained 12.5 μl of 2× PerfeCTa multiplex qPCR supermix, QuantaBio (VWR, Radnor, PA), optimal concentrations of oligonucleotides (Table 1), and PCR grade water and DNA (5 μl). Amplification was carried out with the following cycling conditions: 1 cycle of 95°C for 10 min and 40 cycles of 94°C for 15 s and 60°C for 1 min.

**TABLE 2:** Quadriplex reactions for identification of 20 *emm* types of iGAS commonly found in the United States

*emm* typing reaction	*emm* types^*a*^
1	*emm1*, *emm89*, *emm12*, *emm82*
2	*emm28*, *emm92*, *emm77*, *emm4*
3	*emm59*, *emm11*, *emm3*, *emm2*
4	*emm87*, *emm81*, *emm118*, *emm6*
5	*emm75*, *emm49/151*,^*b*^ *emm76*, *emm83*

a Each target assay identifies known subtypes tested within the *emm* type.

b *emm49/151* is considered here as one type. *emm151* is a rarely encountered 11-codon deletion derivative of *emm49* that occurs within the same MLST type as *emm49* (ST433).

We also designed a synthetic DNA plasmid (Text S1) (Thermo Fisher Scientific; GeneArt), as described elsewhere (14–16), that was used as a positive control for all real-time PCRs. The synthetic DNA was designed to contain primer and probe binding regions for all 20 *emm* types, S. pyogenes-specific target *spy* as an internal positive control, and the *Erwinia* xeno assay for laboratory contamination control, all concatenated in a single plasmid (14).

## RESULTS

The sequential real-time PCR scheme consisted of five 4-plex reactions to identify 20 *emm* types, inclusive of all known subtypes within each type (see ftp://ftp.cdc.gov/pub/infectious_diseases/biotech/tsemm/ for a current listing of *emm* subtypes). Optimal concentrations of both primers and probes (Table 1) were determined for each assay. Monoplex real-time PCR of DNA from 106 GAS isolates from the 20 *emm* types showed that the assays specifically amplified all subtypes assigned to the 20 *emm* types, with the caveat that the *emm49* reaction coidentified the closely related deletion derivative of *emm49*, *emm151* (Table 1). No amplification was observed for the additional 66 *emm* types or for any of the 4 GAS *emm* nontypeable isolates or the 44 non-S. pyogenes species used to challenge the specificity of the assays. When 4 assays were multiplexed together in 5 individual reactions, we also observed no cross-reaction with any of the other *emm* types/subtypes (Table S1) or the non-S. pyogenes species. The multiplex reactions did not produce any false-positive or false-negative results. Among 209 clinical sterile-site GAS isolates (from ABCs) used in the validation, the real-time PCR *emm* typing assays correctly identified respective *emm* types and had 100% concordance with conventional PCR and sequencing and/or WGS results. While we only tested the subtypes for the 21 *emm* types (including *emm151*) that were available within ABCs, we infer based on *in silico* alignment of all available subtypes in the *emm* database for the primer and probe designs that the PCR assays would detect all known subtypes.

The sensitivity of the real-time PCR assays was determined with 10-fold serial dilutions of DNA extracted from all 21 positive-control isolates used in the validation. All newly validated assays had high sensitivity of detection (<10 genome copies/assay) in both monoplex and multiplex formats.

## DISCUSSION

The CDC’s Streptococcus Lab serves as a reference laboratory for streptococci and assists clinical and state public health laboratories by providing support and characterization of GAS, particularly from LTCFs and SNFs, where outbreaks among residents commonly occur (2, 3, 17, 18). Traditionally, PCR combined with sequencing (5) along with other genotyping methods, such as pulsed-field gel electrophoresis (PFGE) and multilocus sequence typing (MLST), have been extensively used to study the clonal relatedness of GAS isolates for epidemiological investigations (3, 17). Recently, WGS has been used for higher discriminatory genetic analysis using single nucleotide polymorphisms (SNPs) to increase resolution in GAS outbreak investigations (2, 18). While all these methods provide valuable information, they either are costly or take several days to complete, and many use equipment and reagents not readily available in most laboratories. The real-time-PCR-based *emm* typing method developed in this study has an advantage over other methods in that it is more rapid, with results generated in a few hours rather than days. Our method identifies four *emm* types in a single reaction and utilizes a sequential multiplex-approach which does not require post-PCR steps for confirmation, saving time and cost. Furthermore, it employs real-time PCR, which is a technology available in many clinical, public health, and reference laboratories. This approach can provide information on the relatedness of GAS isolates circulating in a particular setting and allows for a more rapid response. We have recently found this assay very useful for typing of GAS disease cluster isolates, and we find this approach quite useful prior to proceeding with higher-resolution genomic sequencing of strains sharing the same *emm* types (our unpublished data). Also, this PCR-based approach could further detect GAS from culture-negative or low-DNA-copy-number clinical specimens employing the real-time PCR for the GAS-specific target gene *spy* (19), in which many specimens would be projected to yield *emm* type information from included *emm* type-specific targets.

In summary, a sequential quadriplex real-time PCR scheme was developed that was highly sensitive and specific for the identification of the most frequently occurring 20 *emm* types covering ∼93% of iGAS isolates in the United States collected through CDC’s ABCs program in 2015 (10). Also, the current real-time PCR scheme overlaps 60% of *emm* types (18 of 30 *emm* types) included in a GAS vaccine (4, 20) that has good phase I clinical trial data and targets common M serotypes in the United States, Canada, and Europe. Most clinical and public health laboratories routinely isolate and identify GAS but do not perform DNA-based *emm* typing, as the method is labor-intensive. This method will provide typing capability for these laboratories for outbreak investigation support where *emm* typing may be useful to determine genetic relatedness of isolates to help guide response efforts. A limitation of the method is that the real-time PCR assays do not target all known *emm* types and subtypes, and all subtypes within each of the 20 *emm* types targeted were not tested. However, while this method was developed using the most commonly occurring *emm* types in the United States, the assay also encompasses a large proportion of types globally (21–23) and can be adapted for use in any country where these *emm* types are common.

## Supplementary Material

Supplemental file 1
