# Supplementary material for: Sequential Quadriplex Real-Time PCR for Identifying 20 Common emm Types of Group A Streptococcus
Source: J Clin Microbiol. 2020 Dec 17;59(1):e01764-20. doi: 10.1128/JCM.01764-20 (PMC7771451; doi:10.1128/JCM.01764-20)
Supplement: Supplemental file 1 [file JCM.01764-20-s0001.pdf]

SUPPLEMENTAL TABLE 1 *emm* types/subtypes included in validation testing.

***emm* type**	***emm* subtypes**	**No. of isolates**
**1**[a]	1.0, 1.2, 1.3, 1.5, 1.6, 1.8, 1.11, 1.13, 1.14, 1.18, 1.19, 1.25, 1.29, 1.33, 1.49, 1.79, 1.82, 1.98, 1.99, 1.101, 1.102, 1.109, 1.105, 1.117	24
**2**	2.0	1
**3**	3.1, 3.4, 3.19, 3.33, 3.124, 3.128, 3.129	7
**4**	4. 0, 4.19	2
5	5.3, 5.5, 5.6, 5.12, 5.18, 5.102	6
**6**	6.0, 6.4, 6.5, 6.8, 6,.11, 6.19, 6.54, 6.64, 6.68, 6.101, 6.102, 6.103, 6.106, 6.107, 6.111	15
8	8.0	1
9	9.0, 9.2, 9.4	3
**11**	11.0, 11.1, 11.10, 11.16, 11.17, 11.18	6
**12**	12.0, 12.1, 12.2, 12.4, 12.7, 12.8, 12.9, 12.11, 12.18, 12.19, 12.21, 12.32, 12.34, 12.37, 12.40, 12.75, 12.90, 12.91	18
15	15.1	1
18	18.0, 18.7, 18.12	3
22	22.0 , 22.1	2
24	24.8	1
25	25.1	1
26	26.2	1
27	27.0, 27.4	2
**28**	28.0, 28.19	2
29	29.14	1
31	31.8	1
33	33.0, 33.4	2
41	41.11, 41.2	2
42	42.0	1
43	43.4	1
44	44.0	1
48	48.1	1
**49**	49.0, 49.1	2
54	54.1	1
57	57	1
58	58.0, 58.2	2
**59**	59.0, 59.2, 59.3, 59.4	4
60	60.2, 60.5, 60.9	3
63	63.0, 63.2, 63.3	3
67	67.0	1
68	68.1, 68.3, 68.4	3
73	73.0	1
74	74.0	1
**75**	75.0	1

**76**	76.0, 76.4	2
**77**	77.0, 77.4	2
80	80.1	1
**81**	81.0, 81.1, 81.2	3
**82**	82.0, 82.7, 82.8	3
**83**	83.1	1
84	84.0	1
85	85.0	1
**87**	87.0, 87.3, 87.34	3
88	88.2, 88.5	2
**89**	89.0, 89.1, 89.24, 89.28, 89.33	5
90	90.2, 90.5	2
91	91.0, 91.4	2
**92**	92.0, 92.2	2
94	94.0, 94.1	2
95	95	1
100	100.12	1
101	101.0	1
102	102.2	1
103	103.0	1
104	104.0	1
106	106.0	1
108	108.1, 108.2	2
111	111.1, 111.2	2
113	113.2	1
114	114.6, 114.8	2
116	116.1	1
**118**	118.0, 118.11	2
124	124.2	1
**151**	151.1	1
164	164.4	1
165	165.0	1
169	169.3, 169.6	2
170	170.2	1
183	183.2	1
207	207.1	1
216	216.1	1
223	223.0	1
225	225.0	1
227	227.1	1
232	232.0	1
234	234.1, 234.11, 234.8, 234.9	4
238	238.1, 238.2	2
241	241.0	1
stG245[b]	stG245, stG245.1	2

stG485	stG485	1
stG643	stG643	1
stG652	stG652	1
stG6792.7	stG6792.7	1
	Non-typeable GAS (*emm*-negative)	4
Total		209

[a]*emm* types in bold represent those included in the quadriplex real-time PCR scheme

[b]Group G streptococci – *S. dysgalactiae* subspecies *equisimilis*

SUPPLEMENTAL TEXT:

The sequence of synthetic positive control DNA plasmid used in real time PCR for *S. pyogenes* *emm* typing

GTGATGGTAGTCCTAGGGAAGTTCTTCTTGCAGCAAACAATCCCGCAAAAGCGAGATTAGAGAATGCAAT
GTAAGGCGGACAGTGACAATTGTCTCTGTCGAAGATAATGAAAGAGAATTACATAACACAAGGAGAACAT
CTAGACAAAATAGATGAGATCATAGTGATTTAGTCGCAGAATTTGCAAGACTGAAACGGCTTTCAGAATT
CTACCTTCAGCAATACTATATCACTGAGGCAGGTGTATCTATGGAAGAGTAGGTTTGATGCAGAGCAGAA
TAGAGCAAACGAGCTTGAAAGGCTTCTGCTAATGGAGCTGGTAGCTGCTGCATACAACACATTGCTTACT
GAACATGAGAAACTCGGAGATGAGTGATGACCGGAGCGTTTCTACAATAGTGGTAGCGTGAGCACACCAT
ATTGGCTAAACATGGTGAGCTTGCAAACCAAACAGAAGTTAGGGGGGTTTCTGTAGGTTCAGATGCATCA
CTACATAACCGCATTACAGACCTTCTGATTCAGCGCGGAACTCTGAAGAATATAACGCGTTAGTTCAGGA
AAATGAGGGCTCAAGGTAGAACGTGGACTTTGCAAACCAAACAGAAGTTAAGAAATACGATGCATTGACT
AATGAGAATAAGTCTTTAGAAGAGAGAGAGATAACTATTTAAATTAGGCTTTGCAAACCAAACAGAAGTC
CTAAAGGTACAAACGTGAGCGCACCTATATAATTCGCTATGGGATGAAGCAGACAGTAAAGGCAGATGTG
GGAGTGTTAATGCAGAGTTTCCTAGGCAGAAACTGAGAACTTGTTATATCAGGCAGTTAAGGCGAACAGT
AAGAACCCAAATTAAGTGAAGCAGAATTACATGACAAATATTCGAGAAATTAGATAAAGTTGAAGAAGAG
CCCAGAGAAGTAGCCAGCGAATTGGCTGCTTCAGTGCGGAAGAAAGGATTACTCTGAAATAGAAGGAAAG
TGCAAACCAAACAGAAGTTAAGGTGCGGGTCCAGAAGAGAATGTACCGCGGACATACATAGCCAAACTTG
AAGCGGACAGTAACGCGTCTAGCGTTGCAAAGCTATACAACCAAATGCATCTTACAGATAAAAACGGAGT
ATTCGCTTAGAAAATTAAAAACAGGTGAAGTTAGTGCAAGAGCGTTTCCTGGGGAAGCTAATAATGACAG
GTTAACATATGAAGCACGATACAAAGCATGGAATTATAGAAGGACCTTAGATAAGTTTAATACTGAGCAA
GGTAAGACTACGAGATTAGTGCGGAGAATAACGTGTCTACGTATAGCTCTTTTTCTCTTCTTGCAACGCA
CGGAGAATATCTAGAAAGAATAGGTGCAAACCAAACAGAAGTTAAGGTGAGCTACAGGCTGAACATGATA
AGCTATGAGGAGCTATTGGCTGAACCCAGACAGAAGTTAAGGCTGATAACCAATGCAGTAACACAAGGGG
GCGCGCTTCAGAATTTGTTACATGAAATGGAGCACTCGCTACTATTTCTTACCTCAACCGCAACTCATCA
AGGATTTCTGTTACCAATTACTGGTTTCCAAGACATTGTGACGCCGTTTGTTCACGCTTTGTGAACGCCA
GCAAGACGATCCAGTATCCTATCGGGCTGACTGGCTCT
